# Spatial distribution and multilevel analysis of factors associated with child marriage in Nigeria

**DOI:** 10.1093/inthealth/ihac030

**Published:** 2022-05-19

**Authors:** Obasanjo Afolabi Bolarinwa, Abdul-Aziz Seidu, Zemenu Tadesse Tessema, Collins Adu, Olaoye James Oyeleye, Bright Opoku Ahinkorah

**Affiliations:** Department of Public Health Medicine, School of Nursing and Public Health, University of KwaZulu-Natal, Durban, South Africa; Department of Estate Management, Takoradi Technical University, Takoradi, Ghana; Centre for Gender and Advocacy, Takoradi Technical University, Takoradi, Ghana; College of Public Health, Medical and Veterinary Sciences, James Cook University, Townsville, QLD 4811, Australia; Department of Epidemiology and Biostatistics, Institute of Public Health, College of Medicine and Health Sciences, University of Gondar, Gondar, Ethiopia; College of Public Health, Medical and Veterinary Sciences, James Cook University, Townsville, QLD 4811, Australia; Department of Health Promotion, Education and Disability Studies, Kwame Nkrumah University of Science and Technology, Kumasi, Ghana; Department of Demography and Social Statistics, Obafemi Awolowo University, Ile-Ife, Nigeria; School of Public Health, University of Technology Sydney, Sydney, NSW 2007, Australia

**Keywords:** child marriage, Demographic and health survey, mapping, multilevel analysis, Nigeria, spatial analysis

## Abstract

**Background:**

Child marriage among women has become a major threat to the rights of women, especially in low- and middle-income countries. The marriage of girls below age 18 y is a major public and global health challenge. Therefore, this study examined the spatial pattern and factors associated with child marriage in Nigeria.

**Methods:**

The data were sourced from the 2018 Nigeria Demographic and Health Survey. The study included a total of 4283 young women aged 20–24 y. The findings were provided in the form of spatial maps and adjusted ORs (aORs) with 95% confidence interval (CI).

**Results:**

Hotspot areas for child marriage in Nigeria were located in Sokoto, Kebbi, Katsina, Kano, Jigawa, Yobe, Bauchi, Niger, Borno, Gombe, and Adamawa. The prevalence of child marriage in Nigeria was 41.50%. The likelihood of child marriage in Nigeria was high among those currently working (aOR=1.31; 95% CI 1.11 to 1.55) compared with young women who were not working. On the other hand, young women whose partners had secondary education and above (aOR=0.57; 95% CI 0.45 to 0.73) were less likely to report child marriage in Nigeria compared with those whose partners had no education.

**Conclusions:**

The findings of the study indicate that there are several hotspots in Nigeria that need to be targeted when implementing interventions aimed at eliminating child marriage in the country.

## Introduction

Child marriage among women has become a major threat to the rights of women, especially in low- and middle-income countries.^1^ The marriage of girls below the age of 18 y is not only a major public and global health challenge, it is also a developmental challenge as it affects the general well-being and limits the contribution of young women to national socioeconomic development.^2^ About 12 million girls are married before the age of 18 y every year across the globe.^3^ Globally, there are >700 million women alive who married before the age of 18 y.^4^ Of the prevalence of child marriage across the globe, more than half is practiced in South Asia, Latin America and sub-Saharan Africa.^5,6^ Despite legislative and strategic efforts to stop child marriage, it is still a common problem in sub-Saharan Africa^6^ that affects 54% of women, with large disparities among countries.^7,8^

UNICEF has emphatically stated that child marriage is a violation of human rights because it deprives the individuals involved of many opportunities, such as the right to education, health and safety.^3^ It also has adverse effects on the child, especially girls, and future children, leading to an intergenerational cycle of disadvantages.^9^ There is a global consensus to end girl-child marriage, and Target 5.3 of the Sustainable Development Goals (SDGs) is to ‘eliminate all harmful practices, such as child, early and forced marriages and female genital mutilations’ by 2030.^10^ Ending child marriage has the potential to contribute to eight SDGs, including those addressing economic development (SDG 8), gender equality (SDG 5), inclusive and quality education (SDG 4), good health and well-being (SDG 3) and poverty (SDG 1).^10^

Nigeria has ratified major international treaties such as the United Nations, the International Covenant on Civil and Political Rights, the Convention on the Rights of the Child and the Convention on the Elimination of All forms of Discrimination against Women, with some reservations. There also exists the national strategy for the Prevention and Management of Gender-based Violence. In addition to these treaties to eradicate child marriage in Nigeria, several campaigns, child rights bills and ministerial committees have been instituted and passed, yet the rate of child marriage leaves much to be desired. The practice of child marriage is very common among Nigerian women leading to a continuous rise in adolescent pregnancy in the country.^11^ As the most populous country in the sub-Saharan Africa region, Nigeria has the highest number of child brides,^12^ with >3.5 million women married before the age of 18 y. Child marriage has been identified as one of the major causes of high population growth in Nigeria, with an adverse effect on Nigeria's economy.^13^

National efforts and interventions will yield greater results if they focus on the spatial analysis of factors associated with child marriage in Nigeria, which is lacking. Previous studies have identified several factors associated with child marriage among women. These include education status, place of residence, economic status of the household and the number of family members,^14^ religion^15^ and media exposure.^16^

Despite these studies, less attention is being paid to the spatial differences in child marriage and community factors associated with child marriage in Nigeria. Therefore, we analyze the current Demographic and Health Survey data to determine spatial differences, individual and household/community-level factors associated with child marriage among women in Nigeria. Understanding the factors of child marriage in the most populous country with the highest child brides’ rates in the world is of high relevance to local, regional and global understanding of child marriage and highlights areas interventions should target. To our knowledge, this is the first spatial and multilevel analysis of nationally representative, large-scale data on factors of child marriage in Nigeria. Therefore, this study aimed to explore the spatial distribution and factors associated with child marriage in Nigeria.

## Methods

### Data source

The 2018 Nigeria Demographic and Health Survey (NDHS) dataset was used for the study.

The NDHS is a cross-sectional survey that collects data on men, women, and children's health. The data from the study cover a wide range of health topics, including prenatal care visits.^17^ The NDHS is a nationally representative survey that uses a two-stage sampling technique to obtain data from 36 administrative units and the Federal Capital Territory. The survey's primary sampling unit (PSU) is comprised of samples drawn at random from clusters. A total of 4283 young women aged 20–24 y were considered for this study irrespective of their marital status. The entire methodology of the 2018 NDHS has been published elsewhere.^18^ We followed the standards for strengthening the reporting of observational research in Epidemiology.^19^ The dataset can be downloaded from https://dhsprogram.com/data/dataset/Nigeria_Standard-DHS_2018.cfm?flag=0.

### Outcome variable

The age at first marriage, thus <18 or ≥18 y, was the outcome variable in this study. It was captured as a binary variable where those who married aged <18 y were coded as ‘1’ and those who married aged ≥18 y were coded as ‘0’.^13,20,21^

### Independent variables

Individual- and household-level characteristics were examined in our study based on theoretical and practical significance as well as the availability of variables in the dataset.^13,15,21–23^ These variables were grouped into individual and contextual level factors.

### Individual-level factors

The individual-level factors were maternal current age (20, 21, 22, 23 and 24 y), level of education (no education, primary, secondary and above), partner's level of education (no education, primary, secondary and above), working status (not working, working), mass media exposure (yes, no) and ethnicity (Hausa, Yoruba, Igbo, other).

### Contextual level factors

The contextual level factors were wealth index (poorest, poorer, middle, richer, richest), region (North Central, North East, North West, South East, South South, South West), place of residence (urban and rural), sex of household head (male, female), community socioeconomic status (low, medium, high) and community literacy level (low, medium, high). Community socioeconomic status was computed from the occupation, wealth and education of study participants who resided in a given community. Principal component analyses were applied to calculate women who were unemployed, uneducated and poor. A standardized rating was derived with an average rating (zero) and standard deviation. Similarly, community literacy was obtained using the level of education at the community level. Hence, respondents who had attended higher than secondary school were assumed to be literate, while all other respondents were given a sentence to read, and they were considered literate if they could read all or part of the sentence. Therefore, high literacy included respondents who had higher than secondary education or had no school/primary/secondary education and could read a whole sentence. Medium literacy means respondents who had no school/primary/secondary education and could read part of the sentence. Low literacy means respondents who had no school/primary/secondary education and could not read at all.^24^

### Statistical analyses

We employed both spatial and multilevel analyses in this current study.

### Spatial analysis

#### Spatial autocorrelation

Spatial autocorrelation analysis was performed to check whether there is a clustering effect on child marriage in Nigeria. This analysis result gives Global Moran's I value, Z-score and p-value for deciding whether the data are dispersed or random, or clustered. Moran's I value close to positive 1 indicates a clustering effect, close to negative one indicates dispersed and close to zero random. If the p-value is significant and I value is closer to the mean that indicates a child marriage had a clustering effect.

#### Hotspot analysis (Getis-Ord Gi* statistic)

The hotspot analysis tool gives a Getis-Ord or Gi* statistics for a cluster in the dataset. Statistical values like Z-score and p-value are computed to determine the statistical significance of clusters. Results of the analysis with a high GI* value means hotspot areas (high prevalence of child marriage) and a low GI* value means cold spot areas (low prevalence of child marriage).

#### Spatial interpolation or prediction

Spatial prediction is one of the techniques for estimating unsampled areas based on sampled areas. In NDHS, a total of 1400 clusters were selected to take a sample for this area that is believed to be representative of the country. Bayesian prediction methods were used for this study to predict child marriage in unobserved areas of Nigeria.

#### Spatial scan statistical analysis

Bernoulli's purely spatial model was applied to identify primary and secondary clusters of child marriage. A total of 1393 enumeration areas were included in the final analysis. (SatTscan Software Boston, MA, USA) was used for the analysis. First, the dataset was managed as appropriate for SaTScan software. Women who married aged <18 y were taken as cases, and women who married aged ≥18 y were taken as controls. The cluster number, longitude and latitude data were obtained from the GPS dataset. A cluster size of <50% of the population was taken as upper bound. A 999 Monte Carlo replication was used for this study. Based on the above criteria, primary and secondary clusters were identified.

##### Multilevel analyses

A two-level multilevel binary logistic regression model was designed to analyze the individual and household level characteristics associated with child marriage. In the modeling, women were nested inside households, and households were nested within clusters. To account for the unexplained variability at the community level, clusters were treated as random effects. Four different models were fitted. We started by fitting an empty model, model 0, with no predictors (random intercept). Following that, model I contained only individual-level variables, model II contained only contextual level variables and model III featured both individual- and contextual level variables. The results for models I–III are presented as adjusted ORs (aORs) and their corresponding 95% CIs. This signified the level of precision. The Stata command ‘melogit’ was used to fit these models. The log-likelihood ratio (LLR) and Akaike information criteria (AIC) were utilized for model comparison. The best fit model has the lowest AIC and the highest log-likelihood.^25^ We also looked for multicollinearity using the variance inflation factor, which revealed that the independent variables were not collinear. All analyses were weighted to account for over- and undersampling, and the ‘svy’ command was used to account for the survey's complex character, which also helps with generalizability. All statistical analysis was performed using Stata version 16.0 (Stata Corporation, College Station, TX, USA).

## Results

### Sociodemographic characteristics of respondents

A total of 4283 young women aged 20–24 y were included in the study. The prevalence of child marriage in Nigeria was 41.50%. At the individual level, 29.62% of the respondents were aged 20 y. Less than half (46.58%) had no education, while 2141 (49.99%) of the respondents’ partners had secondary education and above. More than half (55.29%) of the respondents were employed and 57.64% were exposed to mass media. At the household/community level, 25.64% of the study respondents were from the poorer households and 42.02% resided in the North-west region. The majority (77.87%) were from a community with low socioeconomic status, while 51.95% were from a community with a high literacy level. All the individual and contextual level factors were significantly associated with age at marriage in Nigeria (Table 1).

**Table 1.** Individual and Contextual characteristics of respondents by age at marriage in Nigeria

Variable (4283)	Weighted frequency	Weighted percentage	Age at marriage		p-value (χ^2^)
Individual level			≥18 y	<18 y	
Age, y					<0.001
20	1268	29.62	25.09	74.91	
21	593	13.85	33.23	66.77	
22	948	22.04	35.74	64.26	
23	793	18.52	39.50	60.50	
24	684	15.98	49.49	50.51	
Educational level					<0.001
No education	1995	46.58	15.52	84.48	
Primary education	576	13.44	27.84	72.16	
Secondary and above	1712	39.98	60.43	39.57	
Partner's level of education					<0.001
No education	1599	37.34	13.73	86.27	
Primary	543	12.67	28.56	71.44	
Secondary and above	2141	49.99	52.79	47.21	
Working status					<0.001
Not working	1914	44.71	30.61	69.39	
Working	2368	55.29	38.79	61.21	
Mass media exposure					<0.001
No	1814	42.36	20.54	79.46	
Yes	2468	57.64	45.86	54.14	
Religious affiliation					<0.001
Christianity	1221	28.51	61.54	38.46	
Islam	3040	70.98	24.62	75.38	
Traditionalist and other	21	0.51	20.81	79.19	
Ethnicity					<0.001
Hausa	2,284	53.32	18.36	81.64	
Yoruba	382	8.91	75.05	24.95	
Igbo	299	6.98	73.99	26.01	
Other	1,318	30.79	43.82	56.18	
Household/community level					
Wealth index					<0.001
Poorest	1050	24.51	16.19	83.81	
Poorer	1098	25.64	21.52	78.48	
Middle	925	21.60	36.38	63.62	
Richer	763	17.82	56.99	43.01	
Richest	446	10.42	73.20	26.80	
Region					<0.001
North Central	569	15.40	47.10	52.90	
North East	860	20.09	21.80	78.20	
North West	1799	42.02	18.84	81.16	
South East	255	5.95	73.69	26.31	
South South	279	6.51	61.55	38.45	
South West	429	10.03	71.70	28.30	
Place of residence					<0.001
Urban	1343	31.37	53.20	46.80	
Rural	2939	68.63	26.87	73.13	
Sex of household head					
Male	3964	92.56	33.92	66.08	
Female	319	7.44	50.18	49.82	
Community socioeconomic status					
Low	3,335	77.87	28.59	71.41	
High	948	22.13	58.16	41.84	
Community literacy level					
Low	2225	51.95	22.28	77.72	
Medium	701	16.38	30.71	69.29	
High	1356	31.67	58.50	41.50	

NDHS, 2018

### Spatial result

#### Spatial autocorrelation

**Figure 1.**
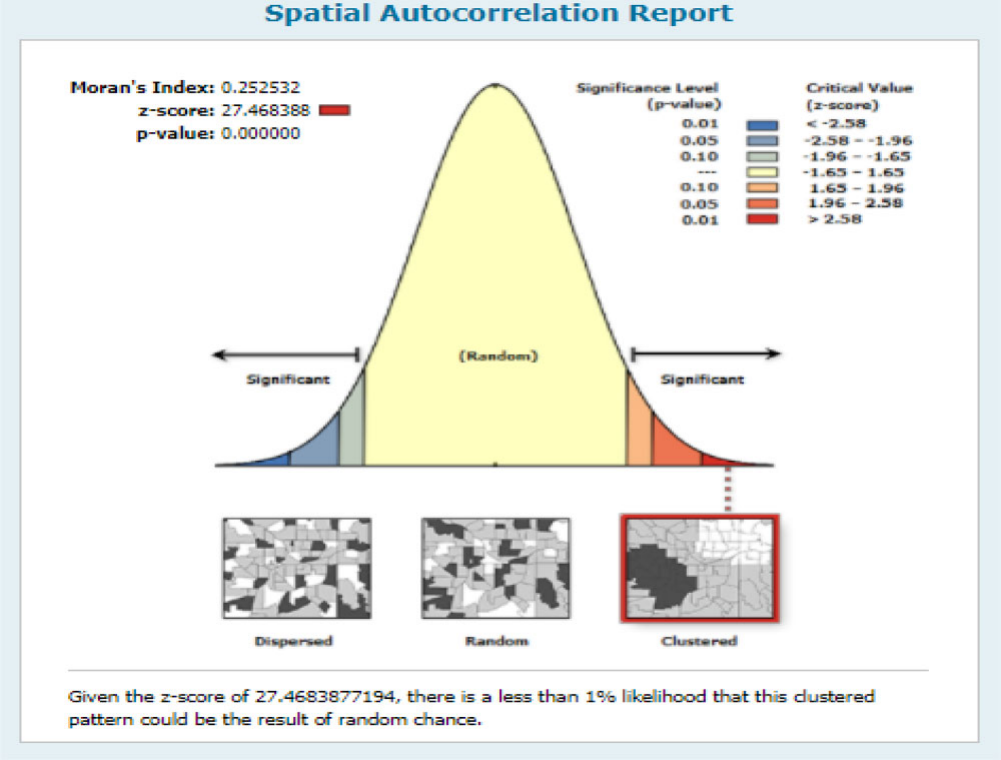
Spatial autocorrelation of child marriage in Nigeria, 2018.

The spatial autocorrelation analysis was performed to check whether child marriage is random or not. According to the Global Moran's I value (0.25), z-score (27.46) and p-value (<0.001), it was found that child marriage was clustered in Nigeria across all the regions (Figure 1).

#### Hotspot analysis

**Figure 2.**
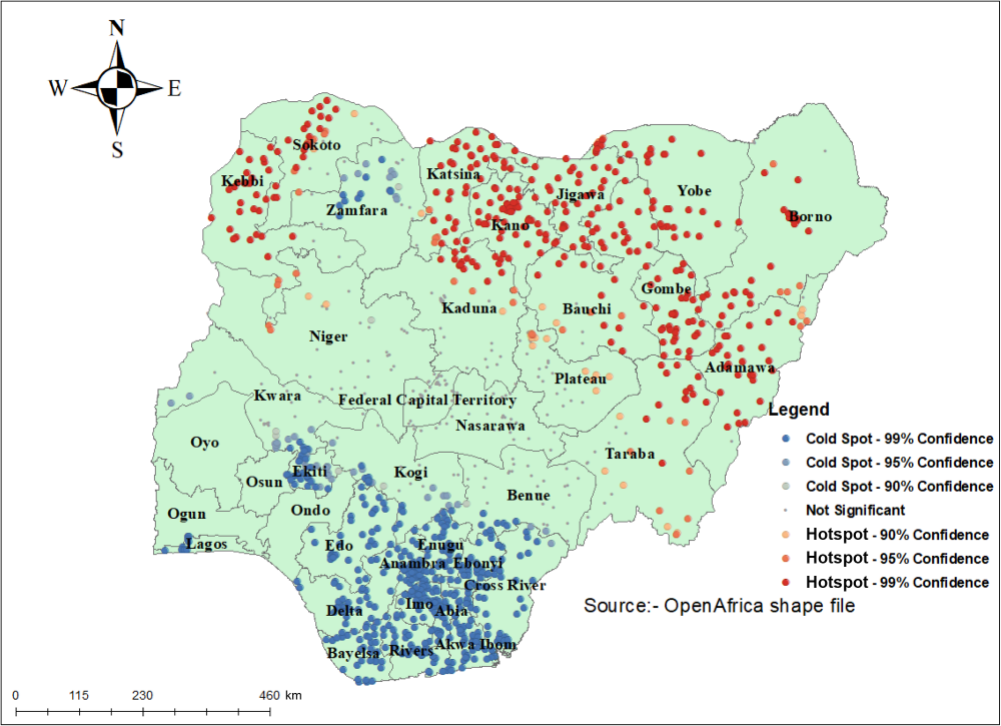
Hotspot analysis results of child marriage in Nigeria, 2018.

Hotspot analysis was performed using Getis-Ord GI* analysis to detect hot and cold spot areas of child marriage in Nigeria. Hotspot areas (a high proportion of child marriage) were located in Sokoto, Kebbi, Katsina, Kano, Jigawa, Yobe, Bauchi, Niger, Borno, Gombe and Adamawa, which are represented by red colors. The cold spot areas (a low proportion of child marriage) were located mostly in parts of Nigeria like Lagos, Osun, Ekiti, Kogi, Enugu, Imo, Abia, Cross River and Zamfara, which are represented by blue colors (Figure 2).

#### Prediction of child marriage

**Figure 3.**
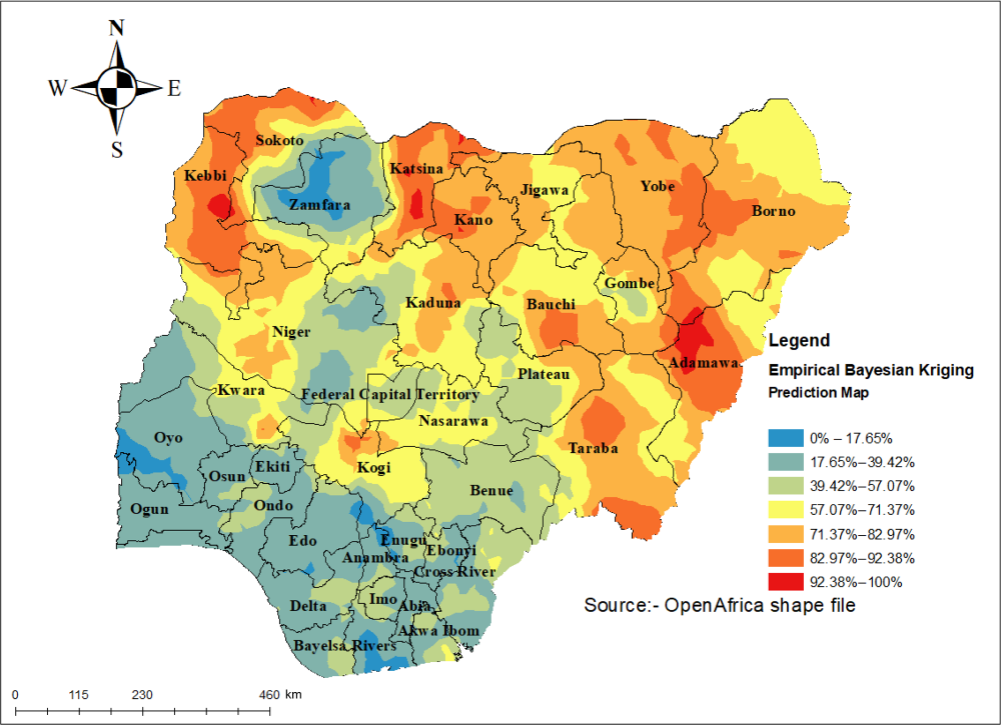
Interpolation of child marriage in Nigeria, 2018.

The prediction analysis was performed and the results show the prevalence of child marriage in unsampled areas of Nigeria based on sampled areas. The prediction revealed that areas shown in red are high-risk areas for child marriage in Nigeria (Figure 3).

#### Spatial SaTScan analysis of child marriage (Bernoulli-based model)

**Figure 4.**
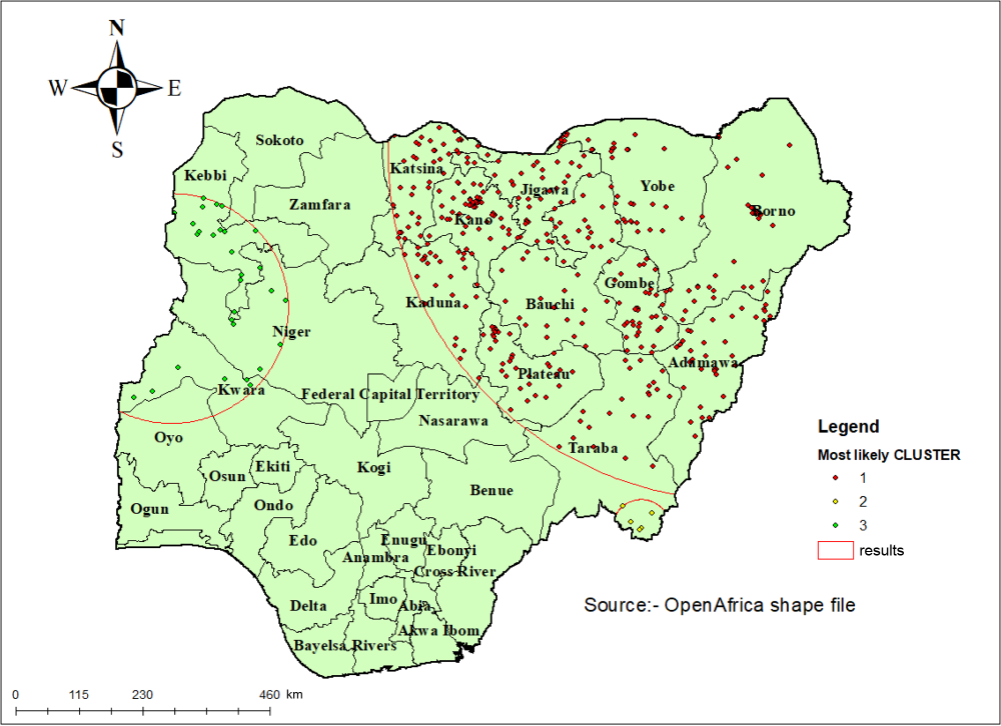
SaTScan analysis results of a child marriage in Nigeria, 2018.

Most likely (primary) and secondary clusters of child marriage were identified. A total of 403 clusters were identified, of which 398 were primary and 5 were secondary clusters. The primary clusters spatial window was located in the northern part of Nigeria (12.719030 N, 12.812300 E)/616.08 km, with a LLR^26^ of 196 and RR 1.56 at p<0.0001. This showed that women within the spatial window had a 1.56 times higher risk of child marriage than women outside the window. Likewise, the secondary clusters were cantered at (6.718072 N, 11.249220 E)/51.39 km radius, LLR of 10.10 and RR 1.50 at p=0.0062. This showed that women within the spatial window had a 1.50 times higher risk of child marriage (Table 2, Figure 4).

**Table 2.** SaTScan analysis results for child marriage in Nigeria

Cluster	Enumeration area (cluster) identified	Coordinate/radius	Population	Case	RR	LLR	p-value
1	398	(12.719030 N, 12.812300 E)/616.08 km	2190	1229	1.56	196	<0.001
2	5	(6.718072 N, 11.249220 E)/51.39 km	32	31	1.50	10.10	0.040

#### Multilevel fixed effects (measures of associations) 

The factors associated with child marriage in Nigeria at the individual level include age, educational level, partner's educational level, working status and ethnicity, while wealth index, region of residence and community literacy level were factors associated with child marriage at the household level (Table 3).

**Table 3.** Multilevel logistic regression models for individual and household/community factors associated with child marriage in Nigeria

Variable Individual-level variables	Model 0	Model I	Model II	Model III
		aOR [95% CI]	aOR [95% CI]	aOR [95% CI]
Age, y				
20		1		1
21		0.90 [0.70–1.15]		0.92 [0.71–1.18]
22		0.80* [0.64–0.99]		0.81* [0.65–1.00]
23		0.67** [0.53–0.84]		0.71** [0.57–0.90]
24		0.52*** [0.41–0.66]		0.56*** [0.44–0.71]
Educational level				
No education		1		1
Primary education		0.86 [0.66–1.11]		0.94 [0.72–1.22]
Secondary and above		0.37*** [0.29–0.46]		0.48*** [0.38–0.61]
Partner's level of education				
No education		1		1
Primary		0.68** [0.51–0.89]		0.72* [0.55–0.96]
Secondary and above		0.48*** [0.38–0.60]		0.57*** [0.45–0.73]
Working status				
Not working		1		1
Working		1.38*** [1.17–1.62]		1.31** [1.11–1.55]
Mass media exposure				
No		1		1
Yes		0.81* [0.68–0.97]		0.95 [0.79–1.15]
Religious affiliation				
Christianity		1		1
Islam		1.22 [0.98–1.53]		1.20 [0.94–1.55]
Traditionalist and other		2.16 [0.93–5.04]		2.02 [0.84–4.84]
Ethnicity				
Hausa		1		1
Yoruba		0.20*** [0.14–0.27]		0.39*** [0.23–0.65]
Igbo		0.25*** [0.18–0.37]		0.41** [0.23–0.74]
Other		0.48*** [0.39–0.59]		0.52*** [0.40–0.67]
Household/community level				
Wealth index				
Poorest			1	1
Poorer			0.91 [0.71–1.15]	1.15 [0.90–1.47]
Middle			0.60*** [0.47–0.78]	0.92 [0.70–1.21]
Richer			0.39*** [0.29–0.52]	0.65** [0.48–0.89]
Richest			0.17*** [0.11–0.26]	0.37*** [0.24–0.57]
Region				
North Central			1	1
North East			2.48*** [1.93–3.18]	1.51** [1.16–1.95]
North West			3.32*** [2.62–4.21]	1.43* [1.06–1.93]
South East			0.49*** [0.34–0.71]	0.86 [0.48–1.55]
South South			0.97 [0.71–1.33]	1.32 [0.95–1.83]
South West			0.57** [0.41–0.78]	0.74 [0.47–1.18]
Place of residence				
Urban			1	1
Rural			0.94 [0.76–1.17]	0.95 [0.77–1.18]
Sex of household head				
Male			1	1
Female			0.70* [0.53–0.93]	0.80 [0.60–1.06]
Community socioeconomic status				
Low			1	1
High			1.31 [0.99–1.73]	1.14 [0.86–1.50]
Community literacy level				
Low			1	1
Medium			0.62*** [0.47–0.80]	0.75* [0.58–0.96]
High			0.46*** [0.37–0.57]	0.73** [0.59–0.90]
Random effects results				
PSU variance (95% CI)	1.96 [1.56–2.46]	0.17 [0.07–0.41]	0.29 [0.16–0.52]	0.14 [0.05–0.41]
ICC	0.37	0.05	0.08	0.04
LR test	χ^2^=333.57, p<0.001	χ^2^=5.94, p<0.01	χ^2^=17.33, p<0.001	χ^2^=4.29, p<0.05
Wald χ2	Reference	749.73***	620.38***	749.21***
Model fitness				
Log–likelihood	−2609.00	−2193.74	−2254.55	−2152.75
AIC	5221.99	4421.48	4541.11	4367.51
Number of clusters	1173	1173	1173	1173

Weighted NDHS, 2018

Abbreviations: AIC, Akaike's information criterion; ICC, intra-class correlation; LR, likelihood ratio; PSU, primary sampling unit.

Exponentiated coefficients; 95% CIs in brackets.

*p<0.05; **p<0.01; ***p<0.001.

Model 0 is the null model, a baseline model without any explanatory variable included.

Model I is adjusted for individual-level variables (age, educational level, partner's educational level, working status, mass media exposure, religious affiliation, ethnicity).

Model II is adjusted for household/community level variables (wealth index, region of residence, place of residence, sex of household head, community socioeconomic status and community literacy level).

Model III is the final model adjusted for both individual and household/community level variables.

At the individual level, currently working women were more likely to have experienced child marriage in Nigeria (aOR=1.31; 95% CI 1.11 to 1.55) compared with young women who were not working. On the other hand, young women aged 24 y (aOR=0.56; 95% CI 0.44 to 0.71), those who had secondary education and above (aOR=0.48; 95% CI 0.38 to 0.61), women whose partners had secondary education and above (aOR=0.57; 95% CI 0.45 to 0.73) and respondents whose ethnicity was Yoruba (aOR=0.39; 95% CI 0.23 to 0.65) or Igbo (aOR=0.41; 95% CI 0.23 to 0.74) were less likely to report child marriage compared with those aged 20 y, those with no education, those whose partner has no education and those from Hausa ethnicity.

At the contextual level, young women currently residing in the North East (aOR=1.51; 95% CI 1.16 to 1.95) and those currently residing in the North West (aOR=1.43; 95% CI 1.06 to 1.93) were more likely to report child marriage compared with those residing in North Central. On the other hand, young women from the richest households (aOR=0.37; 95% CI 0.24 to 0.57) and those from a community with high literacy levels (aOR=0.73; 95% CI 0.59 to 0.90) were less likely to report child marriage compared with those from the poorest households and a community with a low literacy level.

#### Random effects (measures of variations) 

The empty model (Model 0), as shown in Table 3, depicts a substantial variation in the likelihood of child marriage in Nigeria across the PSU clustering (σ2=1.96; 95% CI 1.56 to 2.46). Model 0 indicated that 37% of the variation in child marriage in Nigeria was attributed to intra-class correlation (ICC)^27^ variation (ICC=0.37). The variation between clusters decreased to 5% (0.05) in Model I (individual-level variables only). In the contextual level variables only (Model II), the ICC increased to 8% (ICC=0.08). The ICC declined to 4% (ICC=0.04) in the complete model with both the individual and contextual level factors (Model III). This further reiterates that the variations in the likelihood of child marriage in Nigeria are attributed to the clustering variation in PSUs. Therefore, Model III, the complete model with both the selected individual and contextual level factors, was selected to predict the likelihood of child marriage in Nigeria. The model had the highest log-likelihood ratio and the lowest AIC value. This makes it the best fit model.

## Discussion

This study assessed the spatial distribution and factors associated with child marriage among women in Nigeria using recent national-level data. Nigeria has instituted laws inhibiting child marriage of women, and it is associated with a number of poor physical and social outcomes for young women and their children.^28^ Nigeria has the highest number of child brides^4^ with >3.5 million women married before the age of 18 y. This could be explained by the disparity in cultural differences, educational level and socioeconomic status. This problem needs a collective and comprehensive approach involving religious and traditional leaders, their families, the community and the government, to reduce child marriage and its negative consequences.

The spatial autocorrelation analysis showed that the spatial distribution of child marriage in Nigeria was clustered across all regions of the country. The hotspot analysis showed that child marriage was high in areas like Sokoto, Kebbi, Katsina, Kano, Jigawa, Yobe, Bauchi, Niger, Borno, Gombe and Adamawa. This finding collaborates with previous studies conducted in Ethiopia^20^ and Ghana^23^ that showed regional variations in early marriage. One plausible reason could be that the above-mentioned areas exhibit deep-rooted traditions, such as creating a bond with the bridegroom's family, considering marriage as a success for the girl and her family and ensuring the virginity of the girl before marriage^21^; another important reason for the large variation in child marriage in Nigeria could be that the policies enacted at national level were often ignored at state level.^29^ In this study, as the age of women increases, the likelihood of having a child marriage decreases. One plausible explanation for this could be that young women who married before the age of 20 y were less likely to know about the negative impact of child marriage on their health.^30^ This finding aligns with previous studies conducted in Ethiopia.^20,31,32^

Previous studies have shown that child marriage is often common among poor and less educated communities.^20^ Our study reported a similar finding. Women who had secondary education and above were less likely to marry before the age of 18 y compared with their counterparts with no formal education. Educated women are more likely to have a say in decision-making relating to their family life, the size of their families and the spacing of their children. In addition, most of the years that most women spend in formal education limit their chances of engaging in child marriage. Parents’ roles in the continuity of child marriage are inseparable from their knowledge related to their educational attainment. Parents with less understanding of family life may consider child marriage as the only solution to create better relationships with others.^21,30^

Place of residence is a crucial factor in the pattern of child marriage.^14^ Corroborating the findings of previous studies,^16,33^ we found that women who reside in rural areas were less likely to marry before the age of 18 y. An acceptable explanation for this finding could be that women who reside in rural areas may lack some important education or knowledge about their sexual life, which reduces their chances of getting married.^34^

Akin to the findings of other previous studies,^20,34^ we found that women who were currently working were more likely to marry before the age of 18 y compared with their counterparts. A possible reason for this finding could be that working women are empowered and can afford their needs, making them more likely to marry early.

Mass media exposure (television/newspapers/radio) has a significant impact on marriage age and, therefore, can be used to raise awareness of this issue.^16,35^ Women who were not exposed to mass media were less likely to marry before the age of 18 y. This finding contradicts the findings of other previous studies.^20,34^

The study results show that respondents affiliated with the Hausa ethnic group were more likely to marry before the age of 18 y; this result is in line with a recent study^36^ that reported a higher prevalence of girl-child marriage (54.8%) among the ethnic group. The high rate of early girl-child marriage among the Hausa ethnic group in Nigeria has been linked to the willingness of parents to seal family alliances and keep long-term friendships.^37^

Concerning wealth status, it was noted from the results of our study that women from the richest quintile were less likely to marry before the age of 18 y in Nigeria. This finding contradicts the finding of a joint study conducted in Bangladesh and Ghana.^34^ One possible reason for this could be that women from the richest quintile may have a say in decision-making regarding their relationships and the size of their families. Parents from the poorest wealth quintile see child marriage as a source of reducing the family burden, as they either lure or force their young girls into child marriage because they cannot take care of the needs of those young girls.^38,39^

Women who had secondary and above levels of education were less likely to marry early. This was also similar among those whose partners had secondary education and above level of education.^38^ It is possible that women whose husbands have higher education appreciate the importance of respecting the decisions of their female partners regarding their sexual life and the consequence of going contrary to their spouses’ decisions, reducing the likelihood of child marriage in the country. Also, one plausible reason could be that women whose husbands had higher education spent most of their years in formal education, limiting their chances of marrying early. This finding is consistent with the finding of a previous study conducted in Ethiopia.^20^ Ahonsi et al.^38^ argued that the education of girls was regarded as a protective factor against child marriage.

## Strengths and limitations

The study is limited to variables available in DHS data for analysis. The cross-sectional nature of the data restricts causality. The survey was also based on retrospective self-reports of sampled women, which, as in many surveys, may be prone to social desirability concerns (e.g. recall bias, over and/or under-reporting). Specifically, the definition of child marriage age in this study entertains the possibility of age heaping. Despite these limitations, this study is one of a few that provides a spatial and multilevel analysis of factors associated with child marriage among women in Nigeria that previous research has ignored.

## Practical implications and future research

Current findings underscore the significance of individual and household/community factors as a key indicator of child marriage among women in Nigeria. Women who married early are more likely to come from socially disadvantaged or vulnerable populations, such as those with no or less formal education and no work. Women with these inequalities and in child marriage might be at an increased risk of having partners or husbands who are older and abusive and they are more likely to have restrictions on their mobility and decision-making, including their reproductive health decisions (e.g. consent for sex). Given that SDG4 (i.e. education quality) Target 4.5 advocates the ‘elimination of gender disparities in education and ensure equal access to all levels of education and vocational training for the vulnerable’ and SDG5 (i.e. gender equality) Target 5.3 indicates the ‘elimination of all harmful practices, such as child, early and forced marriage…’,^10^ policies and interventions aiming to raise the age of marriage and providing incentives for girls to stay in school are required. Therefore, the elimination or even reduction of child marriage can have a population-specific impact on women's health and well-being if a greater commitment to the educational development that guarantees retention, continuous support and value for these disadvantaged women is given priority in Nigeria. Additional studies using qualitative and longitudinal designs are required to investigate the factors associated with child marriage, and this could help design appropriate programs to promote women's health in Nigeria. Future studies should also look at the regional pattern of child marriage in Nigeria in relation to the regional differential using spatial analysis.

## Conclusion

The study's findings indicate that there are several hotspots in Nigeria that need to be targeted when implementing interventions aimed at eliminating child marriage in the country. These locations include Sokoto, Kebbi, Katsina, Kano, Jigawa, Yobe, Bauchi, Niger, Borno, Gombe and Adamawa. The government of Nigeria and other governmental and non-governmental organizations need to strengthen policies and programs such as compulsory basic education, poverty alleviation and increased access to media that aim to reduce child marriage in Nigeria. However, most of the interventions should be implemented in the hotspot areas.

## Data Availability

The datasets utilized in this study can be accessed at https://dhsprogram.com/data/available-datasets.cfm.
